# Assessment of a Novel Approach to Identify Trichiasis Cases Using Community Treatment Assistants in Tanzania

**DOI:** 10.1371/journal.pntd.0004270

**Published:** 2015-12-11

**Authors:** Gregory S. Greene, Sheila K. West, Harran Mkocha, Beatriz Munoz, Shannath L. Merbs

**Affiliations:** 1 Dana Center for Preventative Ophthalmology, Wilmer Eye Institute, Johns Hopkins University, Baltimore, Maryland, United States of America; 2 Kongwa Trachoma Project, Kongwa, United Republic of Tanzania; 3 The Wilmer Eye Institute, Johns Hopkins University School of Medicine, Baltimore, Maryland, United States of America; Institut Pasteur, FRANCE

## Abstract

**Background:**

Simple surgical intervention advocated by the World Health Organization can alleviate trachomatous trichiasis (TT) and prevent subsequent blindness. A large backlog of TT cases remain unidentified and untreated. To increase identification and referral of TT cases, a novel approach using standard screening questions, a card, and simple training for Community Treatment Assistants (CTAs) to use during Mass Drug Administration (MDA) was developed and evaluated in Kongwa District, a trachoma-endemic area of central Tanzania.

**Methodology/Principal Findings:**

A community randomized trial was conducted in 36 communities during MDA. CTAs in intervention villages received an additional half-day of training and a TT screening card in addition to the training received by CTAs in villages assigned to usual care. All MDA participants 15 years and older were screened for TT, and senior TT graders confirmed case status by evaluating all screened-positive cases. A random sample of those screened negative for TT and those who did not present at MDA were also evaluated by the master graders. Intervention CTAs identified 5.6 times as many cases (n = 50) as those assigned to usual care (n = 9, *p* < 0.05). While specificity was above 90% for both groups, the sensitivity for the novel screening tool was 31.2% compared to 5.6% for the usual care group (*p* < 0.05).

**Conclusions/Significance:**

CTAs appear to be viable resources for the identification of TT cases. Additional training and use of a TT screening card significantly increased the ability of CTAs to recognize and refer TT cases during MDA; however, further efforts are needed to improve case detection and reduce the number of false positive cases.

## Author Summary

Surgical management of trachomatous trichiasis (TT) is recommended by the WHO as a cost-effective strategy to mitigate blinding trachoma. However, a large surgical backlog exists and many individuals suffering with TT remain unknown to the health system. To identify TT cases, we designed a standard set of screening questions, a card, and simple training for Community Treatment Assistants (CTAs) to identify trichiasis during community-wide Mass Drug Administrations (MDA). To evaluate the sensitivity, specificity, and positive predictive value of this approach, we conducted a community randomized trial in 36 communities in trachoma-endemic Kongwa District, Tanzania. Additional training and the use of a TT screening card increased the sensitivity of TT identification and resulted in more cases identified compared to the usual training of CTAs. The positive predictive value was low, indicating a need for further verification of TT cases identified by the enhanced screening. MDA appears to be a good opportunity for TT screening by CTAs, but further training to improve screening sensitivity is suggested.

## Introduction

Trachoma, the leading cause of infectious blindness, affects an estimated 84 million individuals worldwide [1]. Although eliminated in Europe and the United States, trachoma persists in much of the developing world, disproportionately affecting the poorest and most vulnerable populations [2]. Chronic trachomatous inflammation typically begins during childhood, with years of repeated infections with *Chlamydia trachomatis* leading to the development of conjunctival scarring in a portion of individuals [3–5]. As scarring worsens, an inturning of the lid margin, or entropion, brings the lashes into contact with the cornea. This trachomatous trichiasis (TT), as well as the scarred conjunctiva, leads to damage and opacification of the cornea, ultimately resulting in blindness [6].

To control trachoma and mitigate potentially blinding TT, the Alliance for Global Elimination of Trachoma by 2020 (GET 2020) and World Health Organization (WHO) advocate the multifaceted SAFE strategy, an approach consisting several components: **S**urgery to prevent blindness from TT, **A**ntibiotics to reduce the infectious burden in children, and **F**acial cleanliness along with **E**nvironmental improvement to reduce transmission of *C*. *trachomatis* [7]. As part of the “A” component, WHO recommends mass drug administration (MDA) using oral azithromycin with high coverage, at least 80% of the population in communities where the prevalence of follicular trachoma is >10% [8–10].

Surgical management of TT involves a tarsal rotation procedure, which can be performed by a trained surgical technician within the community [11,12]. However, despite the relative effectiveness of surgery, coverage and uptake are often low due to poor acceptance and awareness of available surgical intervention [13]. Village-based promotional efforts and awareness campaigns have shown success in improving surgical uptake, although identifying TT cases in the community and linking them to services still poses a major challenge to meeting the GET 2020 surgical goal of reducing untreated TT to less than 1 case per 1,000 population at the district level [14–16]. We considered that Community Treatment Assistants (CTAs) providing MDA have the potential to reach all members of a community and may provide a platform for the identification of TT cases.

This community randomized trial sought to develop and test a novel community-based screening approach designed to enhance the ability of CTAs to recognize and report TT cases during MDA. We developed a set of standard screening questions, a TT screening and recognition card, along with additional training. In the randomly selected intervention villages, the approach was introduced immediately prior to MDA, during training of CTAs. Control communities had standard training only. To evaluate the new approach, we compared the number of cases found, as well as the sensitivity and specificity, between the communities with enhanced training group of CTAs and the communities with the control group of CTAs.

## Methods

### Study Design

A controlled community randomized trial design was implemented, using two parallel arms to compare the novel TT screening tool (intervention) to usual care (control) during mass drug administration. The trial was conducted in 36 geographically distinct communities in Kongwa district, Tanzania, between January and November of 2013. Communities were selected based on eligibility to receive antibiotic treatment through MDA.

The community served as the unit of randomization, and enrolled villages were randomly allocated on a 1:1 basis to either intervention (n = 18) or usual care (n = 18) arms.

### Eligibility Criteria

This study was built on to the baseline study of another project, which enrolled 52 communities in Kongwa Tanzania, of whom 36 were to receive MDA. Communities were eligible for this study if they were eligible to receive MDA, and had the consent of village leadership. Within the 36 study villages, individual members of the community were eligible for TT screening if they were 15 years of age or older.

### Mass Drug Administration

In each community, 2–10 CTAs were chosen to distribute antibiotic treatment. CTAs were selected by local leadership based on standing in the community and basic reading skills. Supervisory staff conducted training sessions for CTAs prior to MDA, including how to measure heights of children for antibiotic dosage, to mix liquid doses, to observe treatment provided, to log and manage medication, and to ask about TT and refer self-identified cases of trichiasis for surgery at the local hospital.

### Intervention: Novel Screening Method

In communities assigned to the intervention arm, CTAs received an expanded training session on trichiasis case recognition in addition to the standard instruction on MDA protocol. This additional training lasted one afternoon and consisted of both didactic learning and role-play designed to teach a basic understanding of trichiasis and focusing on four key components:

- What is trichiasis and how does it cause blindness?
- What does trichiasis look like, and what are common symptoms?
- How do you look for trichiasis, using a torch
- What does the surgery involve, and what can one expect after surgery?
- How is an individual added to a referral form, and what will happen after he or she is added?

**Fig 1:**
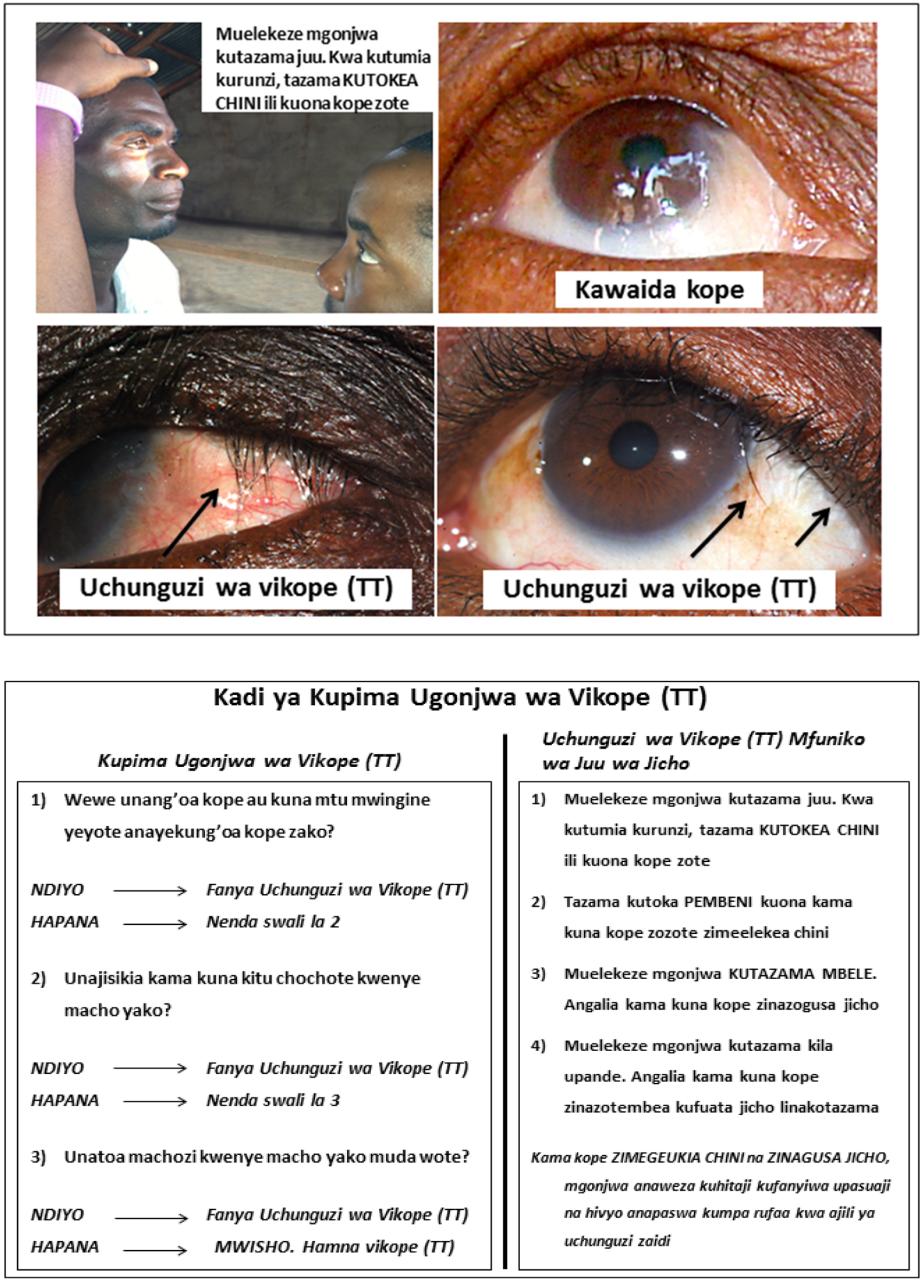
TT screening card.

In addition to this training, CTAs received a TT screening card containing simple questions on symptoms in Swahili, instructions on performing a TT exam, and images depicting proper TT examination and examples of TT cases for reference (Fig 1). Interview questions were designed to identify potential TT cases by asking if an individual had TT (“kope”in Swahili) or had epilated their lashes or had common ocular symptoms of TT, including foreign body sensation and tearing. If a participant said they had TT or epilated, or responded positively to at least one of the three questions, the CTA was directed by the TT screening card to proceed with eyelid examination using a Maglite Solitaire torch as described on the card. Positive cases of TT were defined as individuals who either had one or more lashes touching the globe or epilated. Any suspected TT cases were noted on the treatment log for subsequent verification, at which time they would be provided an opportunity for surgery if indicated.

### Control: Usual Care

In communities assigned to the usual care arm, CTAs received the normal half-day of training on MDA procedures and a half hour basic overview of trichiasis and TT case recognition. CTAs were directed to ask during MDA whether individuals self-identified as having TT, and to record suspected cases of TT on the treatment log for each person. CTAs in usual care communities were given Maglite Solitaire torches identical to those used by CTAs in intervention villages but no other instructions.

### Survey

Following completion of MDA in each village, a survey was conducted by an experienced trichiasis grader, the “gold standard.” He was masked to TT grade assigned during MDA screening. We also attempted to mask him to the village intervention assignment, but once in the village it was difficult to maintain that masking. The follow-up survey was designed to:

- Confirm suspected TT cases screened positive by CTAs
- In a random sample of those screened negative for TT by CTAs, confirm they are truly negative in order to estimate missed TT cases during screening
- Identify TT in a random sample of those who did not attend MDA in order to estimate the number of TT cases missed due to lack of MDA attendance

For the survey, all TT cases reported by CTAs were re-examined to confirm their status. In addition, treatment records were used to randomly select a sample of 100 adults per community initially screened as negative for TT to determine the rate of cases missed by the CTAs during MDA. A random sample of 50 adults per community who did not attend MDA was also selected to estimate the number of TT cases missed because of absence from the MDA.

To conduct the follow-up surveys, invitations were sent to selected individuals following MDA. Invitations notified individuals of the time, date, and location within the community of the survey and requested their attendance. On the day of the survey, the master TT grader examined all presenting individuals for trichiasis. For those found to have confirmed TT, the option of surgical treatment was explained, and interested individuals were added to a surgical referral list for the upcoming surgical camps to be held after the MDA program had concluded. After the initial day of survey, the TT grader continued follow-up on an individual basis until at least 70% participation was achieved for those who had attended MDA.

### Data Collection

CTA screening results obtained during MDA were recorded on paper forms in the mass treatment books. These forms were entered into a customized Access database by staff with double data entry performed for fields of particular importance. All ocular examinations for TT were entered into Samsung Tab 2.0 tablets using a customized ODK electronic form. The ocular exam form included identification information, personal details, and TT status. All electronic data was uploaded into a customized Access database and transferred electronically to Johns Hopkins University for analysis.

### Statistical Analysis

All statistical analysis was conducted using SAS, and Stata version 12 [17–19].

To assess comparability between study arms, differences in demographic and socioeconomic characteristics at the community level were evaluated using Wilcoxson rank sum tests. Additionally, characteristics of participants who were re-examined were compared to those lost to follow up within each arm, using chi-square tests or Fisher’s exact test as appropriate.

Sensitivity, specificity, positive predictive value and negative predictive value were estimated for the intervention and usual care arms using a 2 × 2 table comparing the extrapolated results from the samples verified from the Master Grader with those obtained by the CTAs during MDA, as depicted below in Table 1.

**Table 1:** Sensitivity, specificity, positive predictive value and negative predictive value were estimated for the intervention and usual care arms using a 2 × 2 table comparing the extrapolated results from the samples verified from the Master Grader with those obtained by the CTAs during MDA.

		Master Grader (Gold Standard)		
		**+**	**−**	
Intervention CTA	**+**	A	B	**Total Screened Positive**
	**−**	C	D	**Total Screened Negative**
		**Total True Positive**	**Total True Negative**	

Values used in these calculations as well as in the estimation of disease prevalence were obtained using a simple extrapolation method applying the findings from the random samples in the survey to the study population obtained from the full census of the community. This extrapolation was performed as follows:

1. Proportions of confirmed TT cases in groups initially screened positive by CTAs and successfully followed up with by the master grader were extrapolated to the total number of individuals initially screened positive by CTAs in each arm, included those lost to follow-up (**A** and **B** in Table 1).
2. Proportions of TT cases found in samples of individuals initially screened negative by CTAs and successfully followed up with by the master grader were extrapolated to the total number of negative individuals initially screened as negative in each arm (**C** and **D** in Table 1).
3. Proportions of TT cases found in samples of individuals who did not present at MDA but were successfully followed up with by the master grader were extrapolated to the total number of individuals who did not present at MDA in each arm (used in estimation of prevalence based on total cases found).

A simple extrapolation was deemed appropriate after review of the samples’ representativeness of the respective study population based on age and gender indicators. This method of extrapolation allowed for the comparison of confirmed cases to the estimated total missed cases in both arms, accounting for the rescreening of all individuals initially screened positive and only a sample of individuals initially screened negative.

### Ethics Statement

Oral informed consent was obtained for eligible adults prior to screening. All study protocols and procedures were approved by the Johns Hopkins Institutional Review Board and the National Institute for Medical Research in Tanzania. This randomized trial is registered in the ClinicalTrials.gov database under identifier NCT01783743.

## Results

### Study Participation and Characteristics

**Fig 2:**
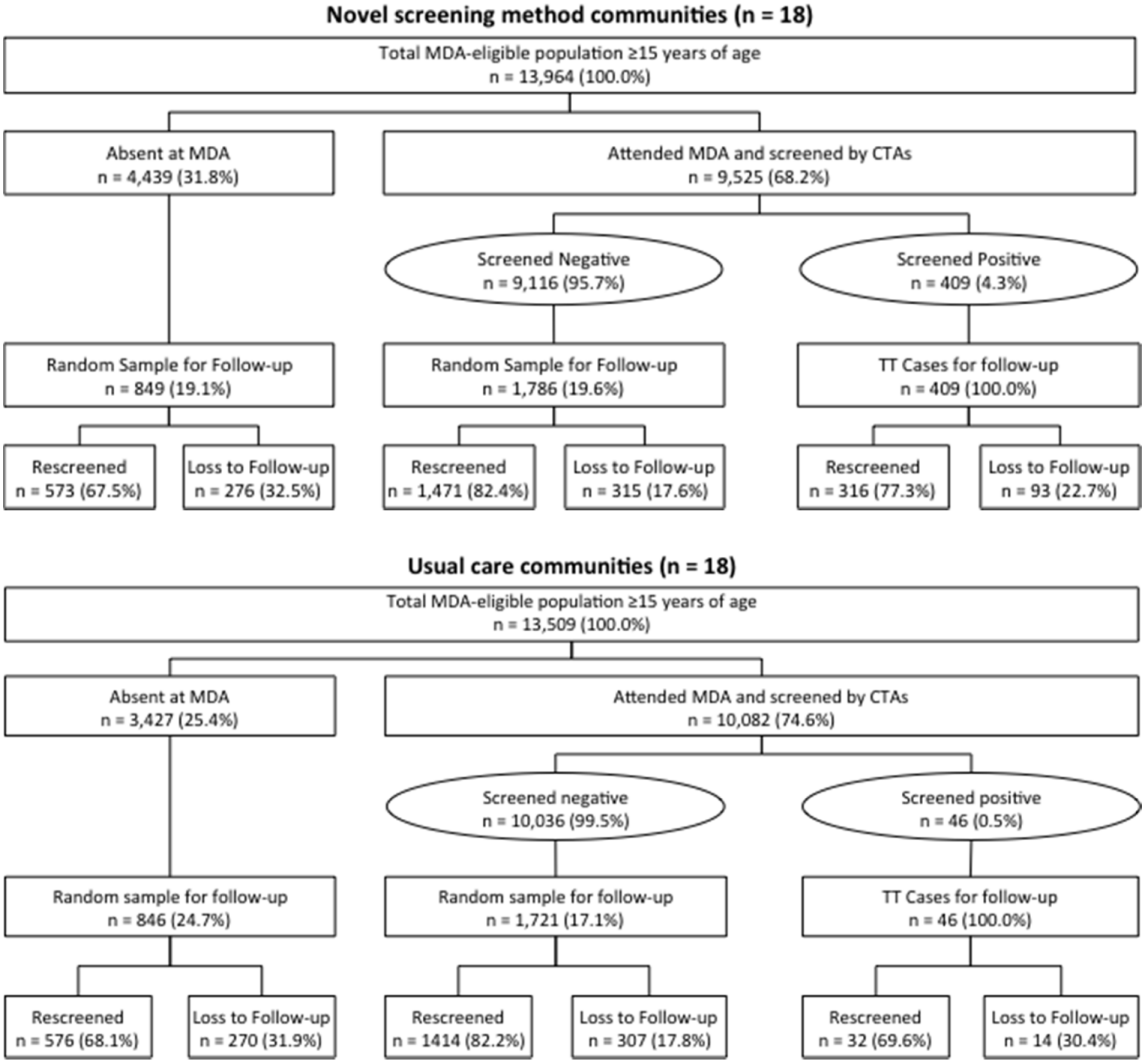
Flow diagram of study design by arm.

A total of 27,473 individuals in the 36 villages holding MDA were eligible for this study, of whom 19,607 (71.4%) attended MDA and were screened for trichiasis. Fig 2 shows the randomization scheme of communities and the flow of participants through the study.

Baseline characteristics of these villages were compared between arms (Table 2), with no significant differences found except a slightly higher proportion of household with bicycles in the usual care arm (median 45% versus 43% in the intervention arm). MDA achieved similar overall coverage in both arms (median 76% versus 72%, *p* = 0.56).

**Table 2:** Characteristics of 36 communities by arm.

	Median (IQR)		p-value*
	CTA Additional Training + Screening Card N = 18	CTA Usual Care N = 18	
*Household Characteristics/ per community*			
Number of households	323 (269–447)	322 (204–460)	0.62
Proportion of households with Latrine	77% (70%–85%)	82% (73%–86%)	0.31
Proportion of households with distance to water>30 minutes	53% (37%–86%)	58% (37%–84%)	0.98
Proportion of households with Bicycles	42% (34%–45%)	45% (42%–53%)	0.04
*Demographic Characteristics/per community*			
Population 15 to 39 years	482 (398–644)	494 (327–682)	0.87
Population 40 to 59 years	152 (119–206)	168 (97–203)	0.85
Population 60 and older	83 (67–108)	81 (54–107)	0.44
Proportion 15 and older who are female	53% (52%–55%)	53% (52%–54%)	0.52
Proportion 15 and older attending MDA	72% (56%–82%)	76% (73%–79%)	0.56

*Wilcoxson rank sum test

CTA = community treatment assistant, MDA = mass drug administration

### Loss to Follow-Up

The master trichiasis grader re-examined at follow up 3,233 (81.6%) of the 3,962 individuals who were initially screened during MDA and who were chosen for follow-up. Additionally, 1,695 individuals who did not attend MDA were randomly selected for follow-up to estimate the number of TT cases missed during MDA. Of these 1695 persons, 1,149 (67.8%) were examined. Loss to follow-up was attributed primarily to refusal or being away from the home, as many residents travel long distances to farmland during the planting and harvest seasons. Table 3 compares characteristics of individuals who were examined by the master TT grader to those who were lost to follow up after MDA. Among the sample who were selected for follow up as screened negative for TT, older males were more likely to be lost to follow up compared to those who were examined. Otherwise, the groups appeared comparable.

**Table 3:** Characteristics of survey groups during follow-up, by arm.

	CTA Additional Training + Screening Card					Usual Care				
	Re-examined		Loss to Follow-up			Re-examined		Loss to Follow-up		
	*n*	%	*n*	%	*p-value* *	*n*	%	*n*	%	*p-value* *
Screened Positive (N = 409, 46)	316/409	77.3%	93/409	22.7%		32/46	69.6%	14/46	30.4%	
Age					0.153					0.543
15–39 years	89/316	28.2%	17/93	18.3%		14/32	43.8%	4/14	28.6%	
40–59 years	94/316	29.7%	30/93	32.3%		9/32	28.1%	6/14	42.9%	
≥60 years	133/316	42.1%	46/93	49.5%		9/32	28.1%	4/14	28.6%	
Gender					0.071					0.277
Female (%)	199/316	63.0%	68/93	73.1%		15/32	46.9%	9/14	64.3%	
Sample of Screened Negative (N = 1,786, 1721)	1,471/1,786	82.4%	315/1,786	17.6%		1,414/1,721	82.2%	307/1,721	17.8%	
Age					<0.001					0.001
15–39	985/1,471	67.0%	176/315	55.9%		1,017/1,414	71.9%	189/307	61.6%	
40–59	269/1,471	18.3%	69/315	21.9%		259/1,414	18.3%	80/307	26.1%	
≥60	217/1,471	14.8%	70/315	22.2%		138/1,414	9.8%	38/307	12.4%	
Gender					<0.001					0.305
Female (%)	856/1,471	58.2%	147/315	46.7%		807/1,414	57.1%	185/307	60.3%	
Sample of Absentees N = 849, 846)	573/849	67.5%	276/849	32.5%		576/846	68.1%	270/846	31.9%	
Age					0.316					0.298
15–39	404/573	70.5%	187/276	67.8%		430/576	74.7%	188/270	69.6%	
40–59	81/573	14.1%	50/276	18.1%		102/576	17.7%	56/270	20.7%	
≥60	88/573	15.4%	39/276	14.3%		44/576	7.6%	26/270	9.6%	
Gender					0.63					0.456
Female (%)	235/573	41.0%	118/276	42.8%		215/576	37.3%	108/270	40.0%	

* χ^2^ test used to compare proportions between re-examined and loss to follow-up groups within each arm.

CTA = Community treatment assistant

### Identification of Trichiasis

After conclusion of MDA, CTAs in the intervention arm reported a significantly higher number of suspected cases (*n* = 409, (4.3%)) than those assigned to usual care (*n* = 46, (0.5%), *p* < 0.01) (Table 4). The proportion of cases confirmed by the master grader as correctly identified was higher in the usual care group (28.1%) compared to the intervention group (15.8%), although this difference did not reach statistical significance (*p* = 0.070). Among those that we could verify, CTAs in the intervention communities identified 5.9 times as many true cases as did those in the control arm (*n* = 50 (0.525%) and 9 (0.089%), respectively), but also had almost ten times as many false positives (*n* = 316 vs. 32). Within the random sample of individuals screened negative by CTAs, the proportions of missed TT cases were not significantly different between the two arms (*p* = 0.269), although fewer cases were missed in the intervention group (1.6%) compared to usual care (2.1%).

**Table 4:** Results of rescreening by master grader.

	CTA Additional Training + Screening Card	CTA Usual Care	*p-*value*
Total Selected for Follow-up	*n* = 3044	*n =* 2613	
Combined Follow-up (%)	2,360 (77.5%)	2,022 (77.4%)	0.895
Screened Positive	*n =* 409	*n =* 46	
* n* selected	409 (100%)	46 (100%)	
* n* examined (%)	316 (77.3%)	32 (69.6%)	0.243
* n* confirmed positive (%)	50 (15.8%)	9 (28.1%)	0.070
Sample of Negative	*n =* 9,116	*n =* 10,036	
* n* selected (%)	1786 (19.6%)	1721 (17.1%)	
* n* examined (%)	1471 (82.4%)	1414 (82.2%)	0.876
* n* TT positive (%)	23 (1.6%)	30 (2.1%)	0.264
Sample of Absentees	*n =* 4,439	*n =* 3,422	
* n* selected	849 (19.1%)	846 (24.7%)	
* n* examined (%)	573 (67.5%)	576 (68.1%)	0.794
* n* TT positive (%)	8 (1.5%)	6 (0.9%)	0.584

* χ^2^ and Fisher's exact tests used where appropriate to compare proportions between arms.

CTA = Community treatment assistant, TT = Trichiasis

The sensitivity, specificity, and positive predictive value (PPV) for both groups were calculated using extrapolated values from the follow-up survey (Table 5). Sensitivity of the additional training and use of the TT screening card (31.2%) was 5.6 times higher than without the extra training and card (5.6%, *p* < 0.05), indicating that the intervention significantly increased the ability of CTAs to identify TT cases. PPV of this screening method did not statistically differ from that of usual care, although both were low (15.8% and 28.1%, respectively). The specificity in the usual care arm was higher (99.7%) than that of the intervention (96.3%, *p* < 0.05), although specificity for both groups was high. Based on case finding and the estimates derived from the random samples, the prevalence of TT in the study communities in those aged 15 and older was estimated to be 2.2% in both arms.

**Table 5:** Community extrapolated sensitivity, specificity, positive predictive value, and negative predictive value by arm: Master grader as the gold standard.

Screening Group	Initial Screening	% Re-examined as Positive	True Positives†	True Negatives†	Sensitivity (95% CI)	Specificity (95% CI)	PPV (95% CI)	NPV (95% CI)	Prevalence in those attending MDA (95% CI)
	A	B	C (A x B)	D (A-C)	**$\frac{\text{C}1}{\text{C}1+\text{C}2}$**	$\frac{\text{D}2}{\text{D}1+\text{D}2}$	**$\frac{\text{C}1}{\text{A}1}$**	**$\frac{\text{D}2}{\text{A}2}$**	**$\frac{\text{C}1+\text{C}2}{\text{A}1+\text{A}2}$**
CTA Additional Training + Screening Card									
Positive (**1**)	409	15.8%*	65	344	31.2%	96.3%	15.8%	98.4%	2.2%
Negative (**2**)	9116	1.6%**	143	8973	(24.9%-37.6%)	(95.9%-96.7%)	(12.3%-19.4%)	(98.2%-98.7%)	(1.9%-2.5%)
CTA Usual Care									
Positive (**1**)	46	28.1%*	13	33	5.7%	99.7%	28.1%	98.0%	2.2%
Negative (**2**)	10,036	2.1%**	213	9823	(2.6%-8.6%)	(99.6%-99.8%)	(15.1–41.1%)	(97.6%-98.2%)	(2.0%-2.5%)

*Percentage of true positive—refer to percentages given in Table 3 –*n* confirmed positive (%)

**Percentage of true negative—refer to percentages given in Table 3 –*n* TT positive (%)

^†^Values extrapolated to the total initial screening group

CI = Confidence interval, CTA = Community treatment assistant

Of the random sample of individuals who did not present to MDA, 14 (1.2%) of the 1,149 individuals examined had TT.

Six months after the follow-up survey, we re-contacted 19 of the 23 TT cases who were screened negative by the intervention CTAs to determine possible reasons for being incorrectly screened during MDA. Four persons were quite elderly and had no recollection of attending MDA or being screened despite mass treatment logs noting their attendance and completion of screening. Of the remaining 15 individuals, 5 (33%) stated that they had not been asked screening questions, 5 (33%) said they had been asked the questions but had not been examined, and 5 (33%) said they had received both questions and examination. When these 15 individuals were asked again the questions using the TT screening card, those who had initially been screened out based on the interview questions (*n* = 5) answered the questions negatively again despite having visible TT. However, those who had not been asked questions during MDA (*n* = 5) and those who had been both interviewed and examined (*n* = 5) answered positively to at least one interview question.

## Discussion

This study demonstrates that an expanded but simple training program and use of a TT screening card improves the ability of CTAs to identify cases of TT during MDA. Using this approach, CTAs identified over five times more TT cases than did those assigned to usual care. Additionally, training and use of the card required minimal additional resources, making this intervention an easily implementable approach to identify TT cases in MDA communities. Although this was a substantial improvement, sensitivity for CTA screening was still lower than expected. As the goal of TT screening is to find those individuals with TT who are currently in need of treatment, a higher sensitivity is necessary to reliably identify as many true positive cases as possible. While the scripted questions were designed to be specific for TT, many individuals answering yes did not have TT. In another study, 43% of false positive cases found by CTAs in fact had other eye pathology, including corneal disease and cataract [20]. From a primary eye care perspective, a high number of false positives may identify other eye conditions that require intervention. Therefore, having an eye care professional undertake further verification would not only help identify TT cases, but could also direct other patients into the eye care system.

During a second follow-up of the false negative cases, one-third again reported not epilating or having symptoms of TT. This may indicate a reluctance of individuals to screen positive for fear of surgery, which has been commonly cited as an issue in TT surgical uptake [13,21–22]. It may also reflect absence of symptoms in some TT cases. Severity data was not collected, so perhaps these individuals had, for example, only 1–2 asymptomatic lashes. Another one-third of the false negatives remember being asked the TT screening questions and examined at MDA; however, they were not correctly identified as having TT by CTA examination. In these instances, the screening questions appear to have proven sufficiently sensitive, and at least three explanations are possible. Perhaps training was insufficient and CTAs failed to recognize TT. Alternatively, it was noted by the program coordinator during MDA that some older CTAs had difficulty seeing at close distances due to presbyopia, which made it difficult if not impossible for them to identify inturned lashes touching the globe. While it would be cost prohibitive to provide loupes for all the CTAs, choosing TT screeners with good near vision should improve detection. Finally, the TT screening card did not stress that epilation could also count as TT and may have led to the false negative cases. While the original intention was to refer patients who epilated or who had lashes touching, if there were no lashes touching the globe due to epilation, the CTAs may not have included the patient in the referral list. The final one-third of the missed TT cases claimed that they were not asked screening questions at all during MDA, suggesting a failure on the part of some CTAs to conduct the TT screening during treatment. All of these individuals responded positively to one or more of the TT screening card questions during their follow-up, raising the possibility that a lack of CTA adherence to the screening protocol may have posed an issue. During field visits, the program coordinator observed that when the MDA became particularly busy, CTAs did not ask questions properly. As CTAs typically work together in pairs during MDA, this could be improved by training CTAs to supervise one another. In fact, as MDA can continue for several years, missed TT cases could be detected in subsequent MDAs.

Both the CTAs in the intervention group and in the control group demonstrated high specificity, which was expected in screening for a relatively rare disease in a population. Maintaining a high specificity is important for this type of screening as correctly identifying individuals without TT reduces the verification need, although as noted, many of these cases may have other eye diseases. The low PPV for TT can be largely explained by the low prevalence of TT. PPVs are typically low in the case of rare diseases for even the most sensitive and specific of tests, as false positives may outnumber true positives [23,24]. Even if expected, a low PPV has significant implications from a programmatic standpoint concerning the planning of surgical services for referred TT cases. Surgical camps, which are commonly implemented in rural and isolated areas, involve a great deal of preparation and cannot be planned for hundreds of cases if only 15% of them actually require surgery [25]. Using our approach, case verification would likely be needed before planning surgical services.

CTAs represent a potential team of screeners already present within endemic communities where TT cases are likely located. Their extensive exposure to the village members during MDA presents a great opportunity for the identification of TT cases. Taking advantage of this exposure for screening comes at only a marginal cost to the MDA itself, requiring only a day of training and the provision of the TT identification card and a torch.

As members of the community, CTAs also possess a deep understanding of the local culture and language, which can be beneficial, particularly when cases are to be referred for surgery. Additionally, charging local leadership with the task of determining recruitment criteria and selection of their own CTAs, as has been implemented in Kongwa, promotes a sense of community buy-in and partial ownership of the trachoma and trichiasis control efforts [26]. Such community-based approaches to disease detection and control also allow for the integration of trichiasis screening into other existing programs. Community-level education programs targeting the Guinea worm have already incorporated mass drug distribution for trachoma control in areas such as South Sudan[27]. Partnerships such as this present opportunity to greatly expand the reach of TT screening while pooling resources to further reduce implementation costs and improve health beyond TT and eye disease.

While use of CTAs in TT screening during MDA offers clear advantages, this approach is limited to areas that merit MDA. This is problematic in areas that have reduced the prevalence of active trachoma to below 5% because even after cases of active trachoma have been reduced, trichiasis cases will likely still exist. Attrition of CTAs may also prove to be a limitation for this screening approach, as significant numbers of community-directed distributors (CDD) in national onchocerciasis programs in Nigeria and South Sudan left after their first year due largely to a lack of incentives offered [27,28]. In our study, both usual care and intervention CTAs participating in MDA were paid a nominal fee for their service, which likely influences retention of these trained assistants in ongoing MDA conducted in Kongwa district. However, this may not be practical for programs that cannot afford to pay CTAs.

In summary, though surgical management of trichiasis has been shown to be cost-effective, the identification of cases needing surgery remains challenging and often resource-intensive. Our study evaluated an innovative approach to TT screening that sought to take advantage of the efforts already being made by trachoma control programs—the use of MDA—as a stage for TT screening. Additional training and use of a TT screening card significantly increased the ability of community treatment assistants to recognize and refer TT cases during MDA; however, further efforts are needed to improve case detection and reduce the number of false positive cases.
